# Newcastle disease virus selectively infects dividing cells and promotes viral proliferation

**DOI:** 10.1186/s13567-019-0644-0

**Published:** 2019-04-18

**Authors:** Zhili Chu, Xiaolong Gao, Haijin Liu, Jiangang Ma, Caiying Wang, Kejia Lu, Qingsong Han, Yanhong Wang, Chongyang Wang, Fathalrhman E. A. Adam, Xinglong Wang, Sa Xiao, Zengqi Yang

**Affiliations:** 1 College of Veterinary Medicine, Northwest A & F University, Yangling, 712100 Shaanxi People’s Republic of China; 2 School of Basic Medical Sciences, Xinxiang Medical University, Xinxiang, Henan 453003 People’s Republic of China; 3 Department of Preventive Medicine and Public Health, Faculty of Veterinary Science, University of Nyala, P.O Box: 155, Nyala, Sudan

## Abstract

Newcastle disease virus (NDV) can select cells to infect, but the mechanism of its cell selectivity has not been comprehensively investigated. Here, we use HeLa cells to establish that NDV can selectively infect cells at the single-cell level. We labeled proliferating cells with 5′-bromo-2-deoxyuridine (BrdU) and examined the colocalization of BrdU with NDV in cells to clarify the relationships between NDV infection and cell proliferation. Receptors at the plasma membrane mediate NDV entry into host cells. We labeled sialic acid receptor isoforms, compared their densities between different cell types and measured the sialic acid receptor densities in different cell phases. Our results suggest that NDV displays host tropism to HeLa cells compared to BHK cells and that the differences in the receptor isoform expression patterns between cell types contribute to the selection of HeLa by NDV. At the single-cell level, the dynamics of receptor expression changes during different cell phases contributing to the selection of cells in S/G2 phase for NDV infection. Furthermore, cell proliferation benefits viral replication, and enhanced virus replication leads to increased damage to cells. The elucidation of the mechanisms underlying host cell selection by NDV may help in the screening and characterizing of additional candidate oncolytic virus strains.

**Electronic supplementary material:**

The online version of this article (10.1186/s13567-019-0644-0) contains supplementary material, which is available to authorized users.

## Introduction

Newcastle disease (ND), which is caused by the Newcastle disease virus (NDV), is one of the most severe avian diseases and can cause great economic loss to the poultry industry worldwide [1]. ND is a contagious disease, and NDV can infect a wide range of domestic and wild birds and kinds of cells. Viruses have evolved to manipulate and take control of the programmed cell death response, but the infected cell attempts to neutralize viral infections by activating different stress signals and defensive pathways to antagonize virus-induced cell self-destruction [1]. Both extrinsic and intrinsic apoptotic pathways can be activated in cells after NDV infection [2], and NDV-induced cell death predominantly occurs via apoptosis [2–5]. As an oncolytic virus, NDV is a promising agent for cancer treatment, and its lytic nature makes it effective in identifying and destroying malignant cells [6]. In the early 1950s, NDV became a promising oncolytic agent [7, 8]. Since then, NDV has been extensively investigated for its use in cancer treatment [9–11]. In 1968, the first attenuated NDV vaccine to be systematically administrated was given to several patients with metastatic carcinoma [12]. To date, NDV has been widely used as a cancer vaccine and an oncolytic agent in several clinical trials of certain human cancers [13–17]. NDV can selectively infect cells, but the mechanism of its cell selectivity has not been comprehensively investigated.

During viral infection, the first challenge that viruses must overcome is gaining access to the intracellular machinery, and the infection process starts when the virus interacts with a target receptor on the host cell, after which the initial steps of virus entry begin [18]. Sialic acids (Sias) represent a family of sugar molecules, with *N*-acetylneuraminic acid (Neu5Ac) as the most common variant [19]. NDV binds to the Sia receptor on host cells and can infect a broad range of cell types and this replication process kills the host cells [20]. However, no study has specifically examined whether NDV selectively infects cells because of differences in the expression pattern of Sia receptors in different cell cycles.

The eukaryotic cell cycle is generally divided into four stages: gap 1 phase (G1), synthesis phase (S), gap 2 phase (G2), and mitotic phase (M); DNA synthesis takes place in the S phase, generating exactly two identical sister chromosomes; G2 phase is a period of rapid cell growth and protein synthesis during which cells get ready for mitosis [21]. In sharp contrast to normal cells that only divide a finite number of times, cancer cells never cease to proliferate. Proliferation is one of the hallmarks of cancer cells, and this perhaps due to the aberrant modification in glycosylation, particularly that in terminal sialylation correlates with tumorigenic transformation and progression [22]. The proliferation activity of cancer cells is often used as a promising therapeutic target for cancer in clinical applications. For cancer cells, it is still not entirely understood whether oncolytic viruses can target its proliferation characteristics and select proliferative cells.

In the present study, by using single-cell and mixed culture experiments, we tested the relationship among host receptors, cell proliferation and NDV selectivity. Our hypothesis was that the cell selectivity of NDV benefits its replication. Dynamic changes in the Sia receptor profile between different cell types and cell phases, along with cell proliferation status, were shown to be important factors responsible for the cell selectivity of NDV.

## Materials and methods

### Animal experiments and ethics statements

The protocol in this study was approved by the Committee on the Ethics of Animal Care and Use of the National Research Center for Veterinary Medicine (Permit 20160313088). All animal works complied with the guidelines of the Animal Care and Use Committee of Northwest A&F University after prior approval.

### Cell culture and virus

The HeLa and BHK-21 (BHK) cells used in this study were stored in our laboratory. BHK-RFP and HeLa-RFP cells were produced by transfecting BHK/HeLa cells with pCDH-CMV-MCS-EF1-RFP before use. All cells were maintained in DMEM (Sigma) supplemented with 10% (or 5%, 2% or 0% where indicated) fetal bovine serum (FBS), 100 U/mL penicillin, 0.1 mg/mL streptomycin, 2 mM l-glutamine (Invitrogen), 1% nonessential amino acids (Invitrogen), and 0.1 mM β-mercaptoethanol (Sigma). The La Sota and F48E9 NDV strains were used in this study. La Sota is a standard genotype II lentogenic NDV strain, and F48E9 is a standard genotype IX NDV strain in China. These two strains were propagated in the allantoic cavity of 9- to 11-day-old SPF embryonated chicken eggs, and all allantoic fluid samples were harvested and stored at −70 °C until further use [23].

### Flow cytometry

For cell cycle analysis, cells were harvested and washed twice with PBS. Cells were digested by trypsin for a relatively long time (5 min), until almost all cells were in a unicellular state. After rinsing, the cells were suspended and fixed with 75% ice-cold ethanol for 24 h at 4 °C. Then, the cells were centrifuged at 600 *g* for 10 min and washed with PBS. Finally, the cells were resuspended in 500 µL of PBS containing 50 µg/mL propidium iodide (PI), 100 µg/mL RNase (MP Biomedicals) and 0.037 mg/mL EDTA (MP Biomedicals) and incubated at 37 °C for 30 min. The prepared samples were analyzed using a FACS (FACSCalibur, BD). Apoptotic cell death was detected by the Annexin V/PI staining assay (HeYan Biotech Ltd., Shanghai) according to the manufacturer’s protocols. Briefly, cells were harvested and washed twice with PBS. Then, the cells were suspended in 400 µL of binding buffer, followed by incubation with 5 µL Annexin V per sample for 15 min. Subsequently, 10 µL of PI per sample was added, and the samples were analyzed via FACS (FACSCalibur, BD, USA). The data were analyzed using FlowJo software (Tree Star, Ashland, OR, USA).

### Immunofluorescence and immunocytochemical analyses

BrdU is commonly used in studies of DNA replication and cell proliferation [24]. To understand whether NDV selectively infects dividing cells, we labeled the cells with BrdU (5 μM) and infected the cells with the La Sota NDV strain (0.1 MOI) for 16 h (La Sota) or 12 h (F48E9). For BrdU immunofluorescence, cell samples were fixed with 4% paraformaldehyde (PFA) for 15 min. After the cells were washed three times with PBS, they were treated with 2 M hydrochloric acid in ultrapure water for 30 min at room temperature, followed by the addition of 0.2% sodium borate solution and another incubation for 30 min at room temperature. Then, the cells were blocked with 1% BSA for 30 min and then incubated with a primary antibody against BrdU (1:100 dilution; BOSHIDE, China) and a chicken polyclonal anti-NDV serum (1:500 dilution; prepared in our laboratory) overnight at 4 °C. The next day, the cells were washed three times in PBS and treated with Alexa Fluor^®^ 488-conjugated goat anti-chicken IgY H&L (1:500 dilution; Abcam, Cambridge, UK) and Alexa Fluor 594-conjugated donkey anti-mouse secondary antibodies (1:500 dilution; Invitrogen, Carlsbad, CA, USA) for 1 h at room temperature in a dark box. Finally, nuclei were stained with Hoechst 33342 (5 µg/mL) for 5 min. Images viewed on a Leica fluorescence microscope or an Andor Revolution WD confocal microscope was captured. For quantification of the GFP/RFP ratio by flow cytometry, the cells were harvested and washed twice with PBS. Chicken polyclonal anti-NDV and mouse anti-RFP primary antibodies (1:300 dilution; Bioss; Beijing; China) were used in this experiment. Fluorescence staining was performed in a test tube, and the staining procedure was performed as previously described [25]. In particular, to wash the cells, centrifugation was conducted five times between each step.

### RNA and q-PCR

Total RNA was extracted with Trizol reagent (Takara, Dalian, China). Single-stranded cDNA was prepared from 0.5 µg of RNA using a reverse transcription kit (Takara, Dalian, China), and specific gene expression levels were then analyzed using q-PCR. The amplification conditions for q-PCR (Gene Star, Beijing, China) were as follows: initial denaturation at 94 °C for 10 min, 40 cycles of 94 °C for 30 s and 72 °C for 60 s, and a final extension at 72 °C for 10 min. The primers used in this study are shown in Table 1.

**Table 1:** **Primer table.**

Name	Forward primer (5′–3′)	Reverse primers (5′–3′)
NDV M gene [26]	AAGAAGCAAATCGCCCC	ACGCTTCCTAGGCAGAG
Human Caspase-3	TGCATACTCCACAGCACCTG	TTCTGTTGCCACCTTTCGGT
Human Caspase-9	AGCAGCAAAGTTGTCGAAGC	TTCTGCTCGACATCACCAAA
Human AKT1	ACTGTCATCGAACGCACCTT	CTCCTCCTCCTCCTGCTTCT
Human Bcl2	GAACTGGGGGAGGATTGTGG	GCCGGTTCAGGTACTCAGTC
NDV-specific reverse-transcription primer [26]	AGGGTTCCCGTTCATTCAG	

### Detection of Sias by lectin staining assay

α2,3 and α2,6 N-linked Sias allow for efficient interaction of NDV with target cells [27]. SNA binds preferentially to Sias attached to terminal galactose through the α2,6 linkage and, to a lesser degree, α2,3 linkage. MAL1 binds to Gal (β-1,4) GlcNAc but tolerates the substitution of *N*-acetyllactosamine with Sia at the 3 position of galactose. After harvesting, cells were fixed with 4% PFA at room temperature for 30 min. Lectin staining was performed with fluorescein-labeled *Maackia amurensis* lectin I (MAL1; Vector Laboratories, San Mateo, CA, USA) and *Sambucus nigra* lectin (SNA; Vector Laboratories, CA, USA) according to the manufacturer’s instructions. MAL1 binds to Gal (β-1,4) GlcNAc but tolerates the substitution of *N*-acetyllactosamine with Sia at the 3 position of galactose, while SNA binds to α2,6-linked Sia. The respective lectins were added to the cell cultures, and the samples were incubated at 37 °C for 30 min and then rinsed three times with PBS. The binding of the labeled lectins to cells was detected using flow cytometry.

### Viral plaque assay

The viral titer was measured by the plaque assay. Briefly, confluent monolayers of BHK cells cultured in 24-well plates were inoculated with cell supernatants at different dilutions (0.1–10 µL per well). After adsorption for 1 h at 37 °C and 5% CO_2_, the liquid was aspirated, and the cells were overlaid with DMEM containing 2% FBS and 1% methyl cellulose and cultured at 37 °C and 5% CO_2_. After 3 to 5 days, the medium was removed, and the cells were lightly washed three times with PBS and fixed with 4% PFA for 30 min. Finally, the plaques were stained with crystal violet. Manual counting of viral plaque and data shown are mean ± SD of three independent experiments.

### Western blot analysis

Proteins were extracted from transfected cells or NDV-infected cells. After the protein samples were boiled for 5 min in 5% SDS–PAGE sample loading buffer, they were separated by SDS-PAGE and transferred to a PVDF membrane for Western blotting. Briefly, the membrane was blocked with 10% skim milk for 12 h at 4 °C. Then, antibodies against BAX, Bcl2, caspase-3 and GAPDH were applied at different dilutions (BAX: 1:500 dilution; Bcl2: 1:500 dilution; caspase-3: 1:500 dilution; and GAPDH: 1:1000 dilution) to detect the proteins. After a 12-h incubation at 4 °C, the membrane was washed three times with Tris-buffered saline containing Tween-20 (TBST). Subsequently, the membrane was incubated with HRP-conjugated anti-rabbit/mouse IgG (1:3000) for 1 h at 37 °C. After four additional washes with TBST, the immunoreactive protein bands were visualized with an ECL Western blotting detection reagent (Bio-Rad), and the results were analyzed using a Tanon-410 automated gel imaging system.

### Statistical analysis

The Student’s *t* test was used when only two groups of data were compared. All data are presented as the means and SD, and the statistical significance of differences is reported as follows: *P < 0.05; **P < 0.01; and ***P < 0.001. All data are representative of no fewer than three different experiments, and the data were analyzed using GraphPad Prism 5 software.

## Results

### At the single-cell level, NDV selectively infects proliferating cells

**Figure 1:**
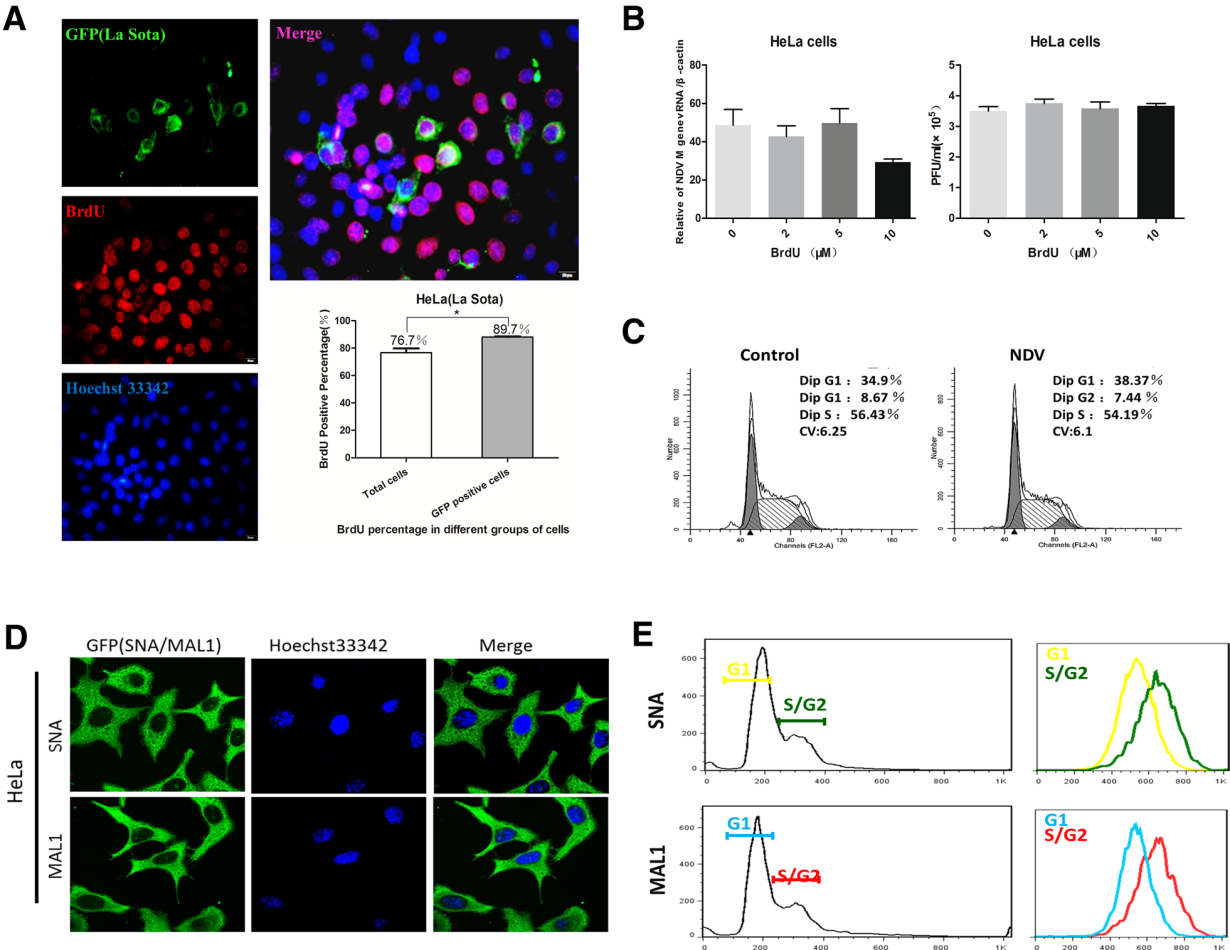
**A lentogenic NDV strain (La Sota) selectively infects proliferating cells. A** Immunofluorescence analysis and quantification of NDV and BrdU staining in HeLa cells infected with the lentogenic NDV La Sota strain (0.1 MOI) and labeled with BrdU (5 μM) at 12 hpi. Scale bar, 20 μm. Data are presented as the mean ± SD of triplicate samples from three independent experiments. *P < 0.05 by t-test. **B** qRT-PCR analysis of NDV M gene expression normalized to β-actin expression in HeLa cells infected with different viral titers and treated with different concentrations of BrdU (0, 2, 5 and 10 μM). Data are presented as the mean ± SD of triplicate samples from four independent experiments. **C** Flow cytometry for cell cycle analysis of HeLa cells infected with NDV (0.1 MOI). **D** Fluorescein-labeled SNA and MAL1 were used to detect Sia expression on the surface of HeLa cells. **E** FACS analysis of HeLa cells colabeled with PI and either SNA or MAL1.

To study whether NDV can selectively infect proliferating cells, we investigated the NDV infection frequencies for HeLa cells in vitro. To further determine the differences between infected and uninfected cells, we used the La Sota NDV strain. Fluorescence staining revealed an inhomogeneous distribution of GFP (NDV)-positive HeLa cells after 16 h of infection with the La Sota virus (0.1 multiplicity of infection: MOI); however, the cell density rapidly decreased around the cluster (Additional file 1). This phenomenon might be due to the adherent growth characteristic of the cells such that the cells around the cluster had more space to proliferate. At 16 h post-infection (hpi), the numbers of BrdU-positive cells and NDV-positive cells were examined by immunofluorescence staining. In HeLa cells (Figure 1A), a proportion of the cells showed dual-positive NDV(GFP) and BrdU(RFP) staining, some cells showed single-positive staining, and some cells showed dual-negative staining. Next, we statistically analyzed the proportion of BrdU-positive cells among NDV-positive cells and the total cell population. The results show that in HeLa cells, 89.7% of the NDV-positive HeLa cells are BrdU positive, while only 76.7% of all cells are BrdU positive (Figure 1A). We then tested the influence of BrdU on NDV replication, and the relative q-PCR results suggest that BrdU had no significant effect on the total viral RNA transcript levels at 16 hpi (Figure 1B). Additionally, the 50% tissue culture infective dose (TCID 50) and viral plaque results show no significant differences among the four groups. By contrast, cell proliferation was inhibited when we infected the cells with NDV (0.1 MOI; 24 h infection) (Figure 1C), suggesting that NDV infection may not enhance the proliferation of infected cells. It has been reported that Sia receptors mediate NDV entry into host cells [27]. We hypothesized that differences in the density of Sia receptors between different cells may correlate with the cell selectivity of NDV. We used fluorescein-labeled SNA and MAL1 to detect the Sia receptor on the surface of HeLa cells. Under confocal microscopy, we found that both SNA and MAL1 binding was strongly positive in HeLa cells (Figure 1D). To determine whether NDV is able to selectively infect cells during a specific phase of the cell cycle at the single-cell level, we labeled the cells with either SNA or MAL1 and PI and then used flow cytometry to detect the Sia receptor density according to the cell cycle stage. The results suggest that the Sia receptor density is higher during S/G2 phase than during the G1 phase (Figure 1E), which may explain why NDV selectively infects actively proliferating cells.

**Figure 2:**
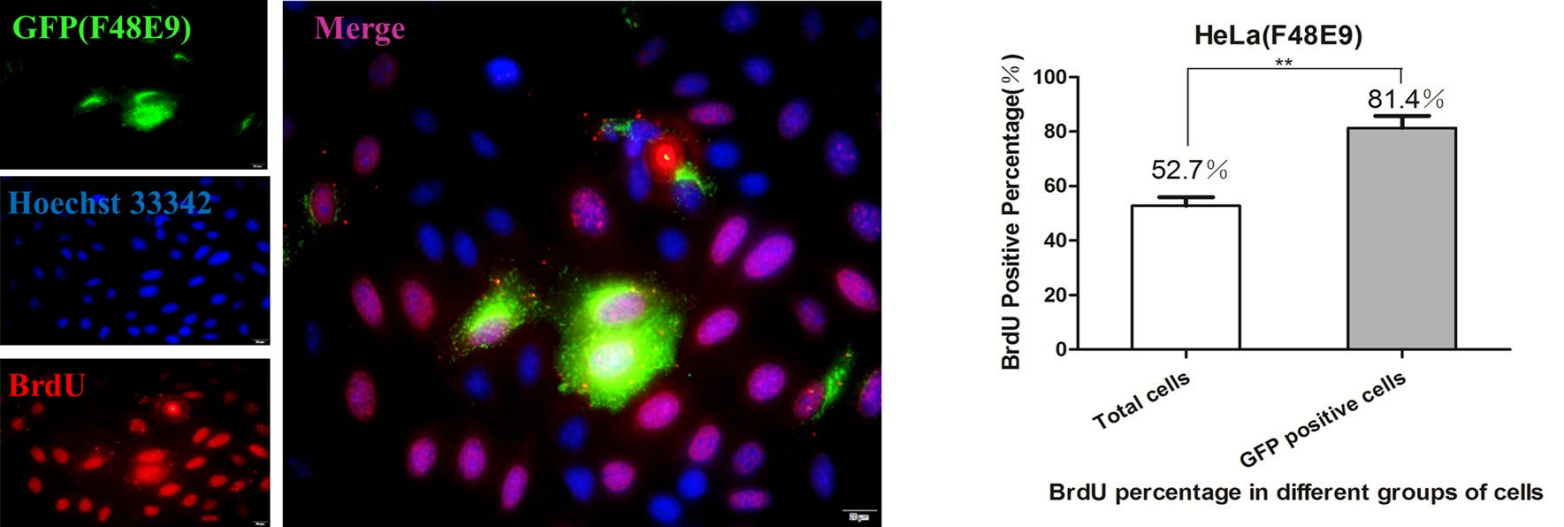
**A virulent NDV strain (F48E9) selectively infects proliferating cells.** Immunofluorescence analysis and quantification of NDV and BrdU staining in HeLa cells infected with the virulent NDV strain F48E9 (0.01 MOI) and labeled with BrdU (5 μM) at 12 hpi. Scale bar, 20 μm. Data are presented as the mean ± SD of triplicate samples from three independent experiments. **P < 0.01 by t-test.

Some NDV strains have been reported to be oncolytic viruses [28]. To understand whether NDV strain differences affect the selection of proliferating cells, we performed experiments with NDV F48E9 strain. For the virulent NDV strain F48E9 which can induce cell fusion post-infection, we inoculated cells with a low viral titer (0.01 MOI) and used the same BrdU dosage as that in the above experiments. At 12 hpi, we verified the results by immunofluorescence staining. Among the treated HeLa cells, 81.35% of NDV-positive HeLa cells were BrdU positive, and only 52.74% of all HeLa cells were BrdU positive (Figure 2). Based on these results, we conclude that NDV selectively infects growing cells independent of the viral strain.

### NDV selectively infects HeLa cells rather than neuraminidase-treated HeLa cells

**Figure 3:**
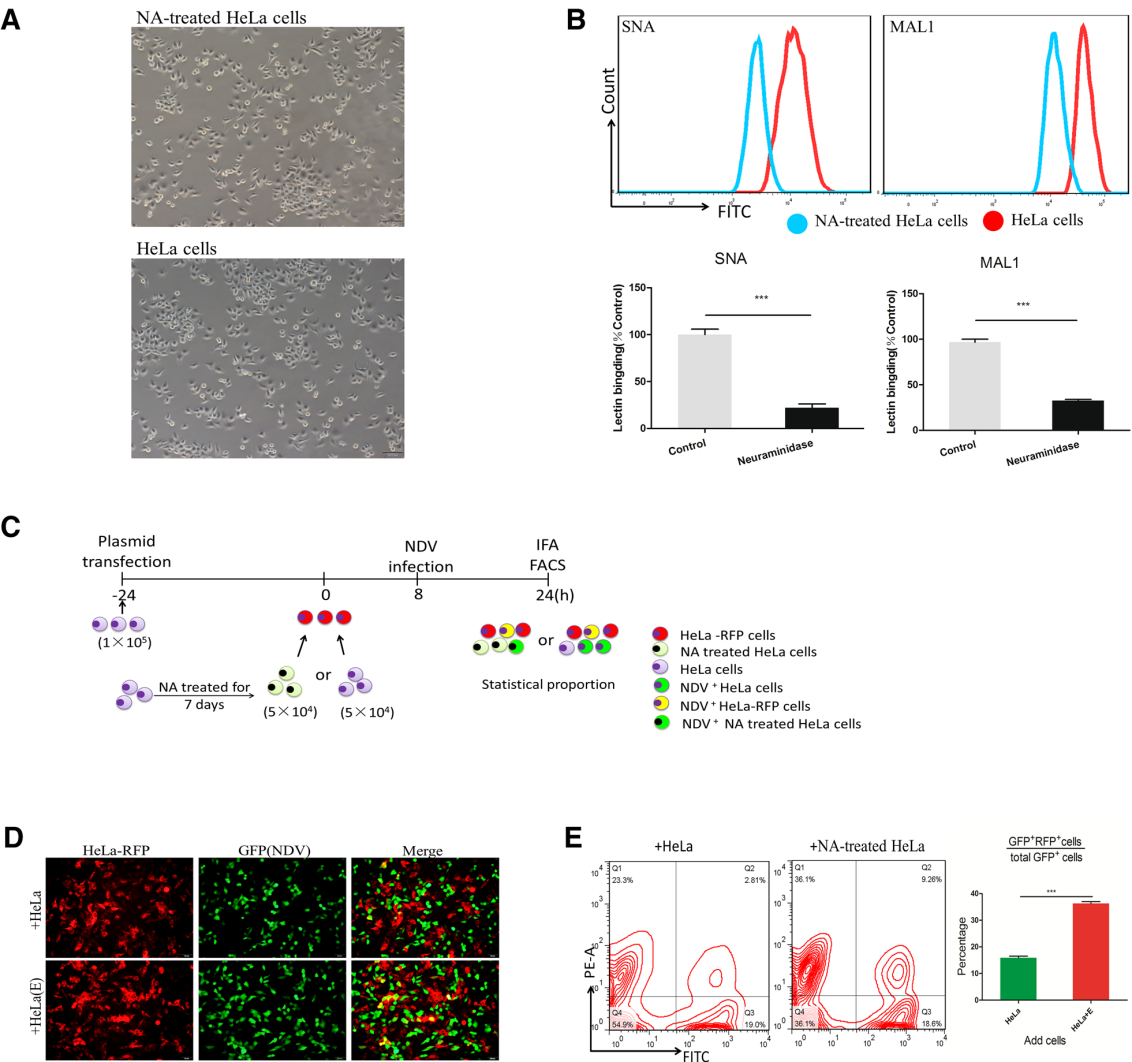
**NDV selectively infects HeLa cells but not neuraminidase-treated HeLa cells. A** Morphologic observation of untreated and neuraminidase-treated HeLa cells. **B** The presence of sialic acid was detected in neuraminidase-treated HeLa cells by FACS analysis; cells treated without neuraminidase were used as controls. Data are presented as the mean ± SD of triplicate samples from three independent experiments. ***P < 0.001 by t-test. **C** Schematic of the coculture assay. **D** Fluorescence microscopy images of NDV (GFP) and RFP expression in cocultured cells. Scale bars represent 50 μm. **E** Flow cytometry analysis of RFP and GFP expression in HeLa-RFP/HeLa and HeLa-RFP/neuraminidase-treated HeLa cells. Numbers indicate the percentage of cells. *n *= 3 per group of four analyses. Data are presented as the mean ± SD of triplicate samples from three independent experiments. **P < 0.01 by t-test.

To determine if Sias were necessary for the selection of NDV, we treated HeLa cells with neuraminidase (N7790, Solarbio, China) that could remove Sias. The cells were incubated with a concentration of neuraminidase (10 μg/mL) for 7 days, and the extent of α2,3- and α2,6-sialylation was assessed using the specific carbohydrate-binding lectins MAL1 and SNA, respectively. There were no cell morphological (Figure 3A) or growth (Additional file 2) differences between untreated HeLa and neuraminidase-treated HeLa (HeLa(E)) cells. Treatment with neuraminidase dramatically blocked the expression of α2,3- and α2,6-linked Sias significantly (Figure 3B). Next, we measured the difference of infectivity between HeLa and HeLa(E) cells by coculture assays. At 24 h post-transfection, different HeLa cells (at a 1:1 ratio) were cocultured with red fluorescent (RFP)-expressing HeLa cells for 8 h and then infected with NDV (0.5 MOI). This was followed by immunostaining for NDV and RFP protein and quantification of the percentage of NDV^+^RFP^+^ (GFP^+^RFP^+^) cells in the NDV^+^ (GFP^+^) cell population by flow cytometry (Figure 3C). The fluorescence results suggest that in the mixed culture system of HeLa and HeLa-RFP cells, NDV-infected cells without bias (the arrow indicates NDV-infected HeLa-RFP cells). However, in the mixed culture system of HeLa-RFP and HeLa(E) cells, NDV more frequently infected the HeLa-RFP cells (more GFP^+^RFP^+^ cells in the field of view) (Figure 3D). To further characterize the selectivity of HeLa cells for NDV infection, FACS analysis was used to statistically determine the proportion of infected cells. In the HeLa-RFP and HeLa mixed groups, the percentage of NDV^+^RFP^+^ cells in the NDV^+^ cell population (approximately 15%) was lower than that in the NDV^+^ HeLa-RFP and HeLa(E) mixed group population (approximately 37%) (Figure 3E). The results indicate that Sia is important for NDV infection.

### Despite the presence of BHK cells, NDV selectively infects HeLa cells

**Figure 4:**
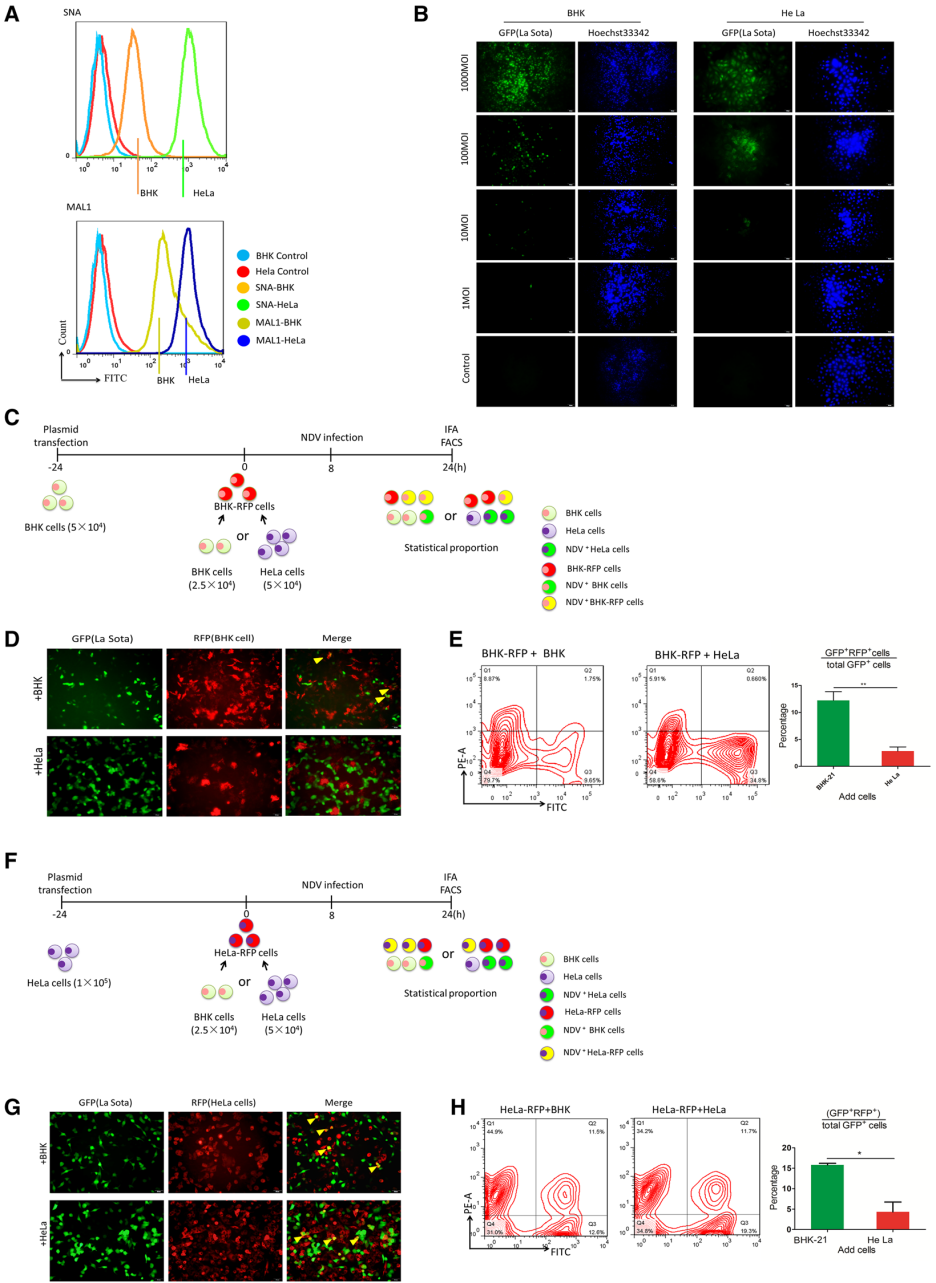
**NDV selectively infects HeLa cells. A** The presence of sialic acid was detected in HeLa and BHK cells by FACS analysis; unstained cells were used as a blank control. **B** Fluorescence microscopy images of NDV (GFP^+^) cells treated with different viral titers of NDV. Scale bars represent 50 μm. **C** Schematic of the coculture assay. **D** Fluorescence microscopy images of NDV (GFP^+^) and RFP in cocultured cells; the arrow indicates an NDV-infected BHK-RFP cell. Scale bars represent 50 μm. **E** Flow cytometry was used to analyze RFP and GFP expression in BHK-RFP/HeLa and BHK-RFP/BHK cell cocultures. Numbers reflect the percentage of cells. Data are presented as the mean ± SD of triplicate samples from three independent experiments. **P < 0.01 by t-test. **F** Schematic of the coculture assay. **G** Fluorescence microscopy images of NDV (GFP) and RFP in cocultured cells; the arrow indicates an NDV-infected HeLa-RFP cell. Scale bars represent 50 μm. **H** Flow cytometry was used to analyze RFP and GFP expression in HeLa-RFP/HeLa and HeLa-RFP/BHK cell cocultures. Numbers reflect the percentage of cells. Data are presented as the mean ± SD of triplicate samples from three independent experiments. *P < 0.05 by t-test.

To further verify the different amounts of Sias on the surface of different cell types, FACS analysis was used to compare the fluorescence intensity among a variety of HeLa and BHK cells (Figure 4A). The fluorescence intensities of both Sia markers were generally higher in HeLa cells than in BHK cells, suggesting that HeLa cells express more Sia receptors for NDV recognition. It is possible that cells were infected by NDV but that the expression of NDV-related proteins was delayed. To explore this possibility, we infected HeLa and BHK cells with La Sota (0, 1, 10, 100 and 1000 MOI) for 6 h and conducted fluorescence staining. As illustrated in the fluorescence images, the numbers of GFP-positive cells (NDV-positive) profoundly decreased with the reduction in NDV titers. The replication of NDV occurred earlier in BHK cells than in HeLa cells even at the same virus dose (Figure 4B). At 16 hpi, aside from GFP-positive cell division or migration, there was no increase in the number of GFP-positive cells (data not shown), suggesting that at 16 hpi, almost all viruses in the infected cells had initiated transcription and replication. It has been reported that the La Sota strain of NDV kills cancer cells in vitro with high selectivity [29], and we hypothesized that NDV can infect based on Sia expression patterns and can select specific cells in cultures containing different cell types in vitro. To test this hypothesis, we used coculture assays with BHK-RFP cells to measure the difference in NDV selectivity between HeLa and BHK cells. At 24 h post-transfection, different HeLa or BHK cells were cocultured with BHK-RFP cells for 8 h. Considering the difference in cell growth between BHK and HeLa cells (Additional file 2), we added BHK and HeLa cells at a 1:2 ratio and then infected the mixed cultures with NDV (0.5 MOI). This was followed by immunostaining for NDV and RFP protein and quantification of the percentage of NDV^+^RFP^+^ (GFP^+^RFP^+^) cells in the NDV^+^ (GFP^+^) population by flow cytometry (Figure 4C). The fluorescence results suggest that in the mixed culture system comprising BHK and BHK-RFP cells, NDV-infected cells without bias (the arrow indicates NDV-infected BHK-RFP cells). However, in the mixed culture system comprising BHK-RFP and HeLa cells, NDV more frequently infected HeLa cells (Figure 4D). To further characterize the selectivity of HeLa cells for NDV infection, we used FACS analysis to statistically determine the proportion of infected cells. In the BHK and BHK-RFP mixed culture groups, the percentage of NDV^+^RFP^+^ cells in the NDV^+^ cell population (approximately 12%) was higher than that in the NDV^+^ cell population from the BHK-RFP and HeLa mixed culture groups (approximately 3%) (Figure 4E).

**Figure 5:**
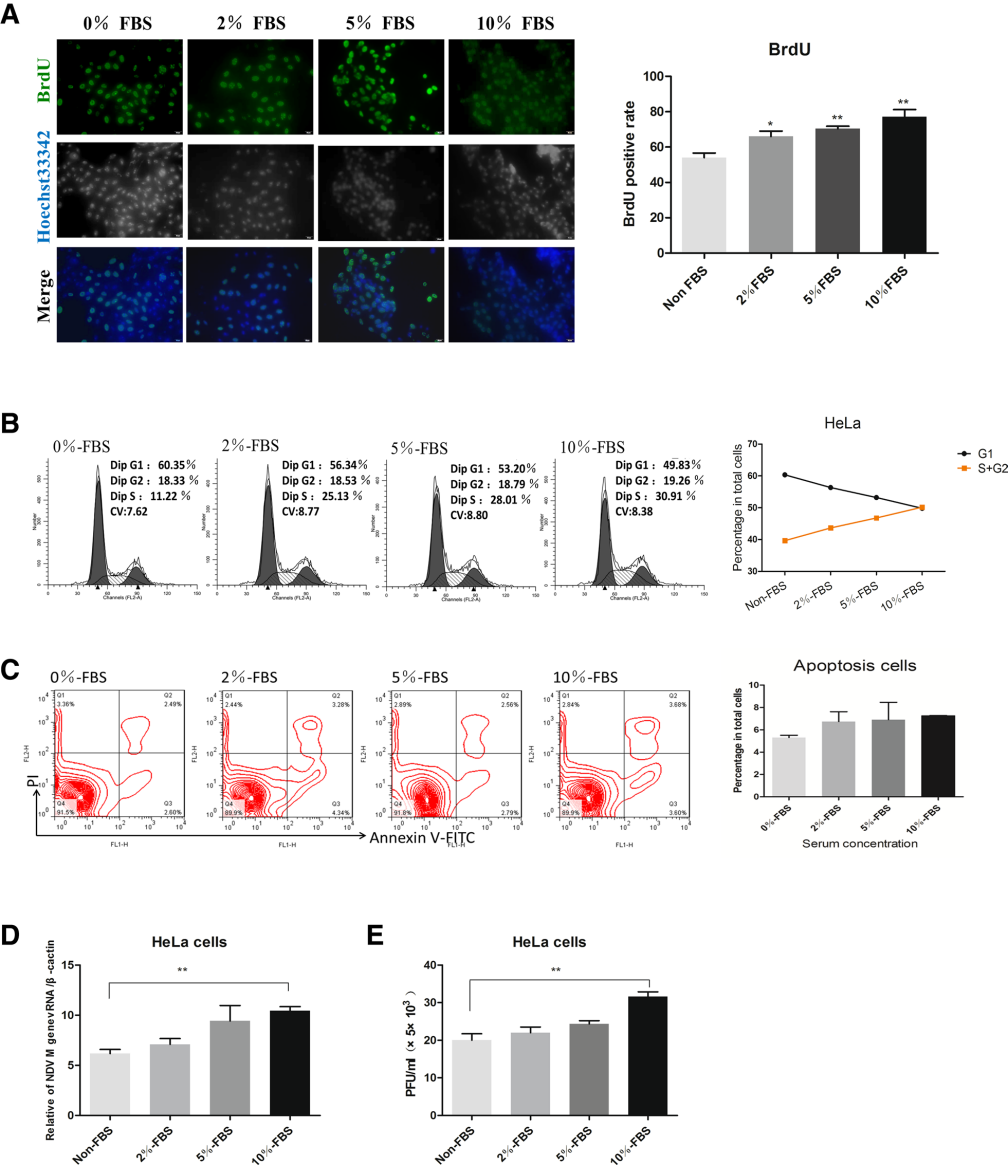
**Proliferating cells enhance NDV replication.** HeLa cells were cultured with different concentrations of FBS for 24 h and then stained with BrdU (**A**), after which cell cycle distribution (**B**) and apoptosis (**C**) were analyzed by flow cytometry. Cells were pretreated with different concentrations of serum for 24 h and then infected with NDV. At 24 hpi, viruses were quantified by qRT-PCR based on M gene expression (**D**) and titration with plaque assay (**E**). Data are presented as the mean ± SD of triplicate samples from four independent experiments. **P < 0.01 by t-test.

Considering that RFP expression in cells may affect the infectious ability of NDV, we repeated the exercise using HeLa-RFP cells similar to the experiment depicted in Figure 5C (Figure 4F). The fluorescence results suggest that in a mixed culture system of HeLa and HeLa-RFP cells, NDV infected the cells without bias (the arrow indicates NDV-infected HeLa-RFP cells, yellow cells). However, in the mixed culture system comprising HeLa-RFP and BHK cells, NDV more frequently infected HeLa-RFP cells (more yellow cells in view) (Figure 4G). FACS analysis shows that in the BHK and HeLa-RFP mixed culture group, the percentage of NDV^+^RFP^+^ cells in NDV^+^ cell population (approximately 15%) was higher than that in the NDV^+^ cell population from the HeLa-RFP and HeLa mixed culture group (approximately 5%) (Figure 4H). This result indicates that NDV displays host tropism for cells that express more Sia.

### Select proliferating cells promote virus replication

To further elucidate whether the selection of proliferating cells by NDV benefits the virus replication, we conducted the following experiments. Given that cell proliferation requires a nutrient supply to control the rate of cell growth, we reduced the concentration of FBS in the culture medium. After incubating the cells in different culture media for 12 h, the cell proliferation index was calculated. BrdU immunofluorescence staining of those cells produced similar results: the proportion of BrdU-positive cells increased with increasing FBS concentrations (Figure 5A). Flow cytometry was used to examine the effects of serum on cell cycle progression, and the results suggest that when the FBS concentration in the culture medium was increased (to no more than 10%), the proportion of HeLa cells in G1 phase decreased (Figure 5B). This result indicates that cell proliferation can be affected by the concentration of FBS. Apoptosis is widely thought to suppress the host cell’s response to NDV replication. In the above experiment, we treated cells with different concentrations of FBS to control the growth rate, but serum starvation might lead to cell apoptosis [30]. We detected apoptosis after changing the medium, and our results suggest that short-term treatment of the cells with medium either lacking FBS or containing a low concentration of FBS for 12 h did not lead to significant apoptosis of HeLa cells (Figure 5C). Therefore, the different replication abilities of NDV under different nutrient conditions appear not to be affected by apoptosis.

To determine the influence of cell proliferation on NDV replication, we infected HeLa cells with the La Sota NDV strain (1 MOI). At 24 hpi, real-time fluorescence q-PCR was used to measure the total viral RNA levels in cell lysates and conditioned media. Compared with the serum-free and low-FBS-supplemented groups, the control group shows higher viral RNA levels in vigorously growing cells (Figure 5D). The plaque test shows similar trends between the F48E9 and La Sota NDV strains (Figure 5E). These results suggest that NDV replication increased with increasing proportions of cells in S and G2 phase, suggesting that selective infection of NDV in dividing cells benefits NDV replication.

### Increased NDV replication leads to more serious damage

**Figure 6:**
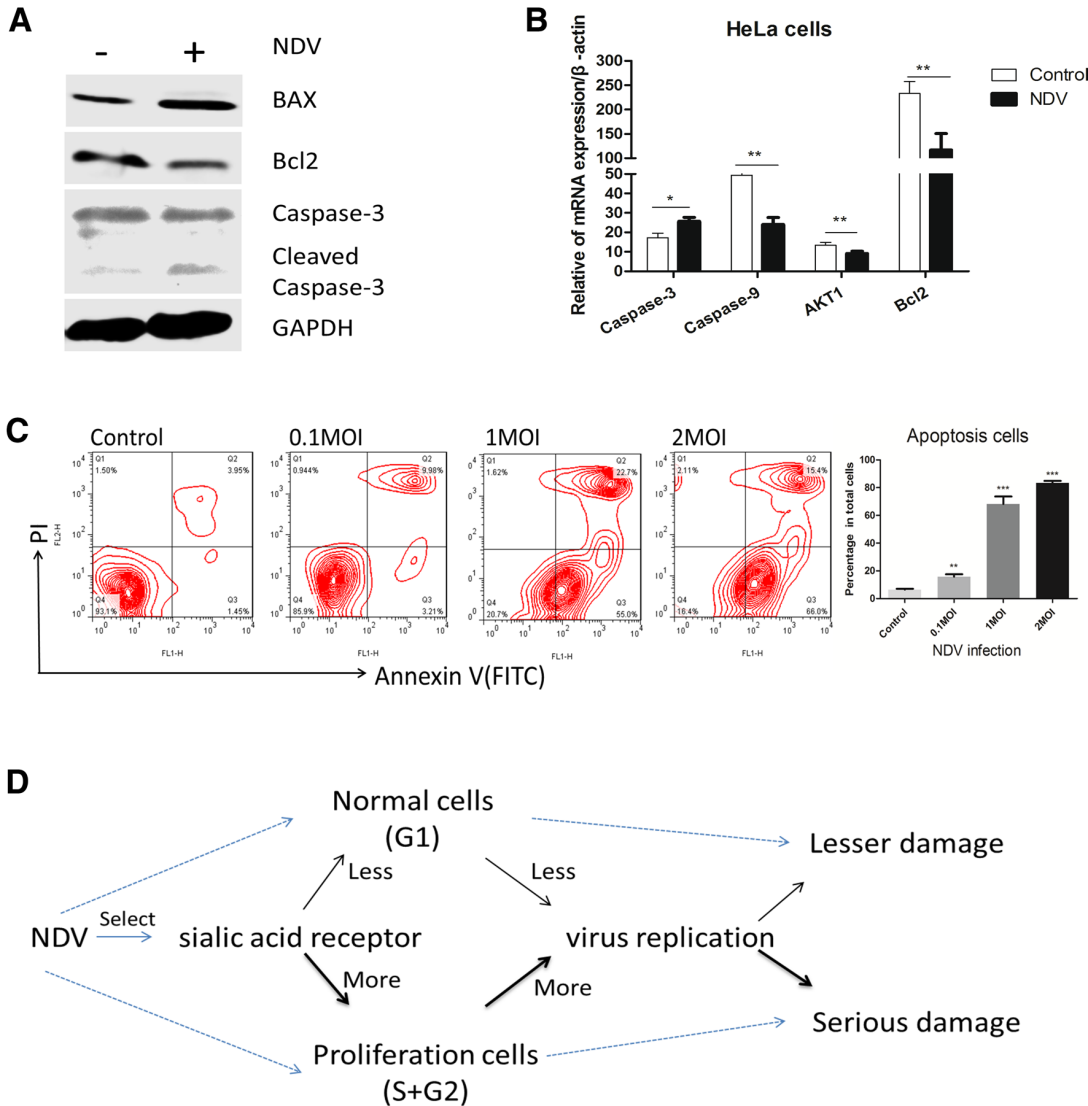
**Increased NDV replication leads to more cell apoptosis. A** Western blot analysis of the expression of apoptosis-related proteins (BAX, Bcl2 and caspase-3) in HeLa cells at 48 hpi (0.1 MOI; La Sota). **B** qRT-PCR analysis of apoptosis-related gene expression in HeLa cells at 48 hpi (0.1 MOI; La Sota). **C** Flow cytometry analysis of cell apoptosis at 48 hpi (0.1, 1 or 2 MOI); data are pooled from replicates of at least three independent experiments. **D** Overall description of how NDV selectively infects proliferating cells to kill tumor cells. Data are presented as the mean ± SD of triplicate samples from a single experiment and are representative of three independent experiments. *P < 0.05, **P < 0.01 and ***P < 0.001 by t-test.

Apoptosis has been identified as a major hallmark of NDV-mediated cytotoxicity in virally infected cells [1]. NDV-induced apoptosis requires virus entry, replication and de novo protein synthesis [31]. Our results suggest that NDV selectively infects growing cells to produce more viral copies, and we speculated that stronger viral replication could more strongly induce apoptosis. To test our hypothesis, cells were infected with NDV (La Sota strain) at different MOI for 48 h. Western blot analysis shows that when cells were infected with the La Sota NDV strain (0.1 MOI) for 48 h, HeLa cells exhibited notable apoptosis based on the changes in the relative expression of apoptosis-related proteins (BAX, Bcl2, cleaved caspase-3) (Figure 6A). The q-PCR results suggest that caspase-3 expression was increased in HeLa cells from the NDV-infected group (0.1 MOI) at 48 hpi compared to that in cells from the control group (Figure 6B). The flow cytometry results were consistent with the hypothesis that NDV infection of the cells at a higher MOI would increase apoptosis (Figure 6C). The cytotoxicity of NDV to cells is known to be due to its ability to activate multiple caspase-dependent pathways of apoptosis [3]. These results indicate that cells with a higher proliferative capacity will produce more virus, while more virus replication will lead to more pronounced apoptosis (Figure 6D).

## Discussion

For the cell selection mechanism of NDV at the single-cell level, we observed that NDV tends to infect cells in the S/G2 phase (Figure 2). To our knowledge, there are no reports about the relationship between the cell cycle and changes in Sia dynamics. Our findings suggest that cells in the S/G2 phase have a higher receptor density than do cells in the G1 phase (Figure 2), which may explain why NDV selectively infected BrdU-positive cells in single-cell cultures. No study has reported on differences in cell selectivity between different NDV strains. In our work, both the virulent F48E9 strain and the lentogenic La Sota strain selectively infected proliferating cells, but their potencies in selecting proliferating cells differed (Figures 2, 3). NDV selectively infects dividing cells in HeLa and BHK cell cultures, but the ratios were not exactly the same. Thus, we conclude that the capability of selecting S/G2 phase cells is a common characteristic of NDV that may vary according to the cell type and NDV strain. Based on this method, an evaluation system for screening of the oncolytic NDV strain may be established.

As extensively demonstrated in vitro and in accordance with a classic rule of thermodynamics, the more attachment factors or receptors that are present at the cell surface and the higher the affinity of a virus with these receptors, the more efficient the primary interaction is [32]. It is well known that different cell types express distinct receptor types at specific quantities. In our study, NDV preferentially infected HeLa cells relative to HeLa(E) and BHK cells, and HeLa cells expressed SNA- and MAL1-labeled Sia receptors on their surface more abundantly than did HeLa(E) and BHK cells (Figures 4 and 5); this difference may be why NDV selectively infected HeLa cells. In addition, it has been reported that α2,6-linked Sia functions as a high-affinity receptor with NDV in oncolytic virotherapy [33]. BHK cells lack α2,6-linked Sia (Figure 5A), which may be another reason NDV selected HeLa cells. Normal cells were found to be resistant to NDV replication. Therefore, we investigated the relationship between cell proliferation and viral replication. Our results suggest that cell proliferation is beneficial to viral replication (Figure 6). It has been reported that the nutrient/physical status of the host links innate immunity to antiviral host defense and that a higher nutrient status increases virus replication [34]. In our study, we used different concentrations of FBS to regulate cell growth and found that cells with a higher proliferative ability exhibited increased virus replication. These results suggest that NDV replication may be affected by the nutrient/physical status of the host cell.

Viruses have evolved to manipulate and take control of the programmed cell death response, but the infected cell attempts to neutralize viral infections by activation of different stress signals and defensive pathways to antagonize the virus-induced cell self-destruction [35]. The inherent anti-tumor capacity of NDV combines two characteristics that delineate what can be defined as the oncolytic paradigm: NDV promotes the induction of tumor cell death accompanied by the elicitation of antitumor immunity [1]. Both extrinsic and intrinsic apoptotic pathways can be activated in cancer cells after NDV infection [2], and apoptosis dominantly contributes to NDV induced cell death [2–4, 36]. In lung cancer cells, higher virus particles infecting the cell corresponded to more cell death [37]. In our experiments, apoptosis was more extensive in HeLa cells infected with NDV at a high MOI than in cells infected with NDV at a low MOI (Figure 6). Therefore, we conclude that NDV selects dividing cells for infection and replication, leading to apoptosis of those dividing cells. NDV has the ability to selectively kill apoptosis-resistant cells [38]. In our study, we found that cell proliferation was not positively correlated with cell apoptosis (Figure 6), suggesting that the selection of proliferating cells by NDV is independent of apoptosis resistance. Tumor cells have a lower capacity to resist NDV replication, and another reason may be that tumor cells have a lower interferon level [39, 40] or are resistant to apoptosis [38]. Given the limitations of materials and methods in this study, we did not measure the interferon concentration at the single-cell level. Here, we show the selectivity of NDV among the same cells in one culture dish grown in cell culture medium containing the same interferon concentration, suggesting that the selection of proliferating cells by NDV is independent of the extent of interferon resistance.

In general, our results suggest that in one cell type, NDV tends to infect cells during the S/G2 phase in the absence of interference from other cell types. Moreover, the selective infection of dividing cells by NDV benefited its replication, and enhanced NDV replication in cells with increased cell damage (Figure 6D).

## Additional files

**Additional file 1.** **NDVselectively infects proliferating HeLa cells.** (A) Immunofluorescence for NDV (0.1 MOI) revealed an uneven distribution of infected HeLa cells at 16 hpi. Scale bar, 50 μm. (B) Immunofluorescence for BrdU revealed an uneven distribution of infected HeLa cells. Scale bar, 50 μm.

**Additional file 2.** **Cell growth curve.** Cell growth curve of neuraminidase-treated HeLa (HeLa + E), HeLa and BHK cells.
