# Supplementary figures and images for: Newcastle disease virus selectively infects dividing cells and promotes viral proliferation
Source: Vet Res. 2019 Apr 18;50:27. doi: 10.1186/s13567-019-0644-0 (PMC6472075; doi:10.1186/s13567-019-0644-0)

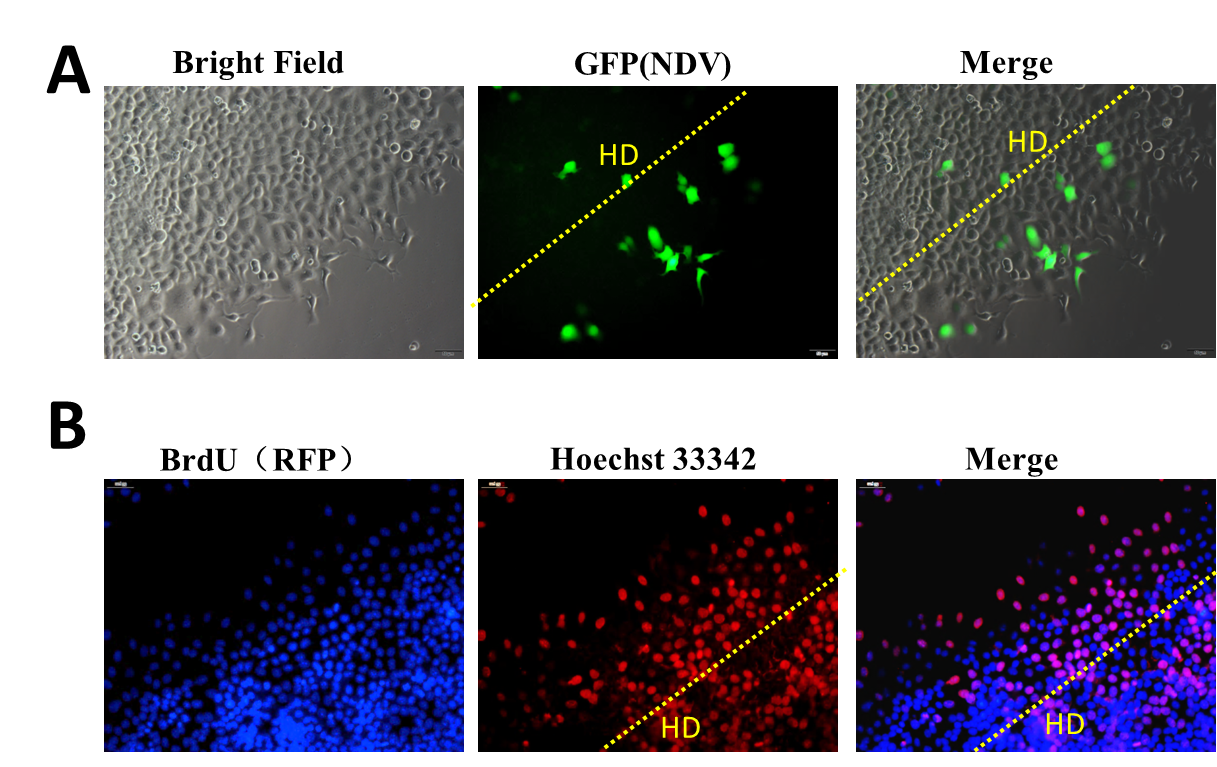

Supplement: Supplementary file 1 — Additional file 1. NDVselectively infects proliferating HeLa cells. (A) Immunofluorescence for NDV (0.1 MOI) revealed an uneven distribution of infected HeLa cells at 16 hpi. Scale bar, 50 μm. (B) Immunofluorescence for BrdU revealed an uneven distribution of infected HeLa cells. Scale bar, 50 μm. [file 13567_2019_644_MOESM1_ESM.tif]

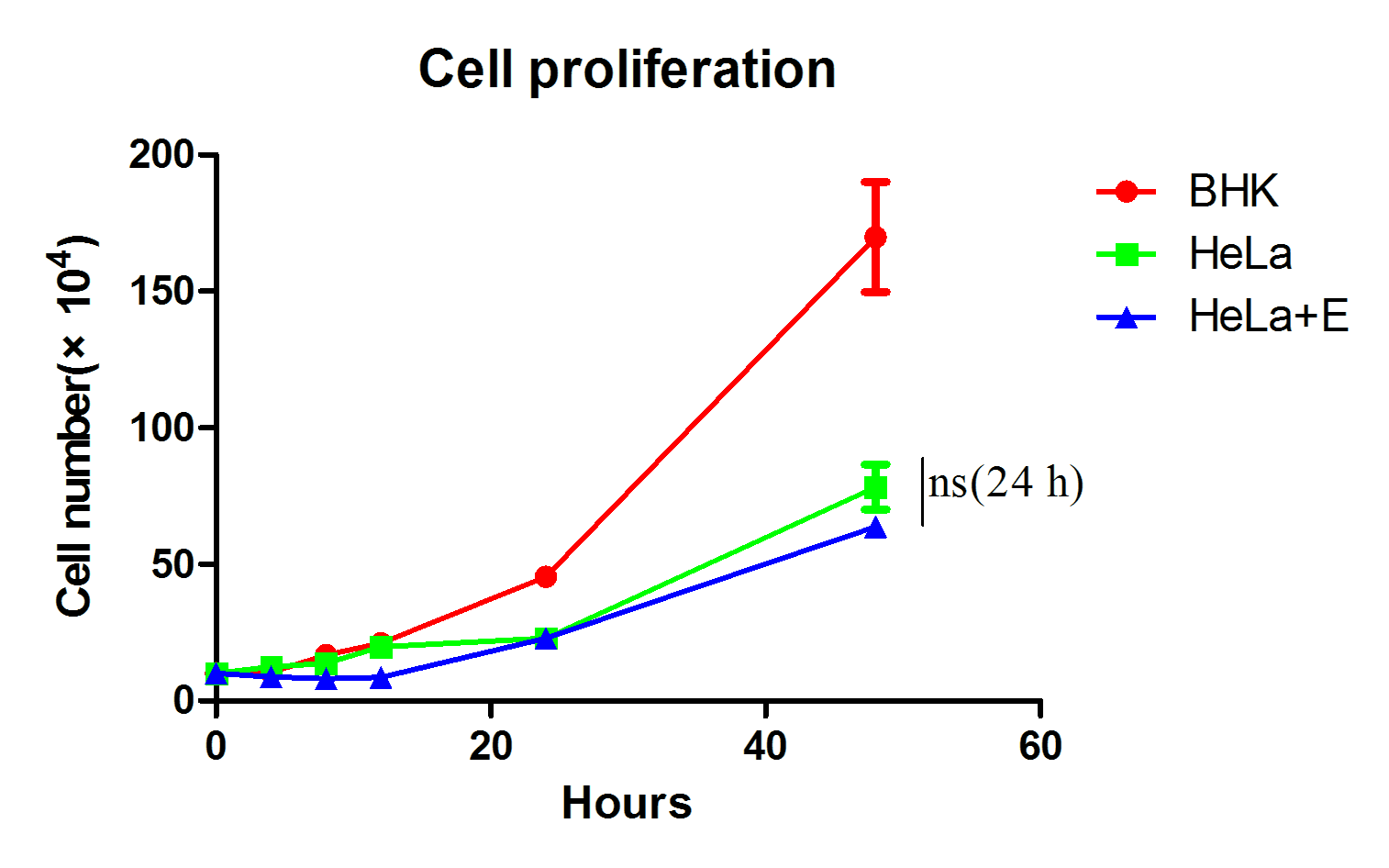

Supplement: Supplementary file 2 — Additional file 2. Cell growth curve. Cell growth curve of neuraminidase-treated HeLa (HeLa + E), HeLa and BHK cells. [file 13567_2019_644_MOESM2_ESM.tif]
